# Objectively measured physical activity, physical activity related personality and body mass index in 6- to 10-yr-old children: a cross-sectional study

**DOI:** 10.1186/1479-5868-6-25

**Published:** 2009-05-14

**Authors:** Benedicte Deforche, Ilse De Bourdeaudhuij, Eva D'hondt, Greet Cardon

**Affiliations:** 1 Department of Movement and Sports Sciences, Ghent University, Watersportlaan 2, 9000 Gent, Belgium; 2 Research Foundation Flanders (FWO), Belgium; 3 Department of Human Biometry and Biomechanics, Faculty of Physical Education and Physiotherapy, Vrije Universiteit Brussel, Pleinlaan, Brussels, Belguim

## Abstract

**Background:**

The prevalence and level of overweight in childhood is rapidly increasing. One potential contributor to the rise in overweight is a decline in physical activity (PA). The purpose of this study was to compare levels and patterns of PA and PA related personality in normal-weight (NW) and overweight (OW) 6- to 10-yr-old children.

**Methods:**

Subjects were grouped into OW (N = 59, BMI = 24.2 ± 4.8 kg/m^2^) or NW (N = 61, BMI = 15.7 ± 1.5 kg/m^2^) according to International Obesity Task Force cut-offs. PA was assessed by accelerometry. Parents filled in a questionnaire on PA and sedentary behaviour and PA related personality of their child (born tired, moves slowly, is often tired, lacks energy, avoids physical efforts, prefers watching playing children instead of joining them, is always active, needs to let himself/herself go, has a lot of energy).

**Results:**

NW children spent on average 77 min/day in MVPA, whereas OW children only 57 min/day (p = .001). OW children had fewer 5, 10 and 20 min bouts of MVPA (p = .01). OW and NW children showed identical PA patterns on both week days and weekends, although at different levels. According to parents' report, a greater percentage of OW children was not engaged in any sport (46% versus 23%, chi^2 ^= 6.3, p = .01). OW children had a less active personality (p < .001), watched more TV during weekend (p < .01), but no differences were found in outside play or non-active play. BMI of mother and father explained 29% of the variance in children's BMI z-score (p < .001). PA related personality, screen behaviour during weekend and MVPA explained an additional 12% (p < 0.01).

**Conclusion:**

The results of this study demonstrate that NW children spent on average 20 min per day more in MVPA. PA patterns were similar in NW versus OW children, although at different levels. Greatest differences in PA according to weight status were found in the afternoon during after school hours. This is the first study to show distinct PA related personality traits in OW children compared to NW peers.

## Background

The prevalence and level of overweight and obesity in childhood is rapidly increasing worldwide [1-3]. This is concerning since childhood overweight is associated with psychosocial and medical consequences [4,5]. For the primary and secondary prevention of obesity related diseases, it is important to gain insight into factors related to overweight and obesity in children. One potential contributor to the rise in overweight and obesity is a decline in physical activity (PA). Some studies have shown that overweight (OW) children have lower levels of PA compared to their normal-weight (NW) peers [6]. However, most of these studies rely on self-reported data. The major limitation of self-report is the inability to accurately recall PA, especially that of daily living. Children, in particular, are not cognitively capable of recalling specific activities and their intensity and duration. Therefore self-reports should not be used in children under the age of 10 years [7]. Parents, as proxy-reporters, can provide useful information on PA preferences and patterns of their children, particularly activities occurring during defined periods, but parents can only give a crude estimate of actual PA levels [8]. To reduce the error associated with self-reported methods in children, objective measurement devices such as accelerometers should be used.

Few studies worldwide evaluated accelerometer-derived levels of PA in OW and NW 6- to 10-yr-old children and results of these studies are inconsistent. Some studies have shown that OW young children are less likely to be physically active than their NW peers [9-11], while other studies found no consistent differences in PA according to weight status [12-14]. In a large cross-sectional observational study in 9- to 10-y-old children from four European countries (European Youth Heart Study), PA measured by accelerometry explained only a small amount of the variance in body fatness (<1%) [9].

Besides levels of PA, studying the patterning of children's activity across the day is also important in understanding children's activity behaviour and in designing subsequent PA interventions. Previous studies showed that 'after school hours' is the most active time of the day in children [15] and that the least active children are especially less active during 'after school hours' [16]. However, to date, no previous studies have examined PA patterns in a non-clinical sample of OW young children. Only one previous study compared patterns of PA in clinically obese children with a non-obese control group [10]. They found that differences in activity level according to weight status were most marked when children are more likely to be able to choose to be active, that is, before school, at lunchtime, and particularly after school and in weekends.

Some studies suggest that certain personality characteristics play a role in the development of overweight and obesity in children [17]. Negative mood has been found to predict increased weight gain in infants between 6 and 12 months of age [18] and easily distressed infants who are difficult to soothe have been found to have higher skin fold thickness and lower activity levels than other children at 2–3.5 yrs of age [19]. An explanation for this finding could be that difficult children might be fed more often to quiet them. Maternally rated difficult temperament has also been associated with excess weight gain in middle childhood [20] and temperamental difficulty in childhood has shown to be a risk indicator for general body mass in adulthood [21]. A child's personality can be adequately assessed using parental ratings [22-24]. To the best of our knowledge, there have been no previous studies investigating "PA related" personality traits in relation to overweight in young children. The tendency to be active could be considered an inborn characteristic in the same way as children's willingness to take new foods and accept specific foods has strong-to-moderate heritability [25,26].

Although there is solid evidence that PA plays an important role in the development of overweight and obesity, there is a lack of objective data describing levels and patterns of PA in relation to overweight in young children and no information regarding PA related personality traits. Therefore the purpose of this study was 1) to compare levels and patterns of objectively measured PA and sedentary behaviour between OW and NW young children, 2) to determine whether the PA related personality of OW young children is different from that of NW peers, and 3) to study the cross-sectional association between PA and PA related personality traits and overweight status in young children.

## Methods

### Subjects

A sample of 120 (6- to 10-yr-old) schoolchildren was recruited from ten randomly selected primary schools in East and West Flanders, two Flemish provinces. All parents were informed about the study and when they were interested to participate, they were asked to report stature and body mass of their child to get an indication of their overweight status. When a child was identified as being overweight according to the international cut-off points [27], a classmate with the same gender and age was selected to be a control child. The final sample consisted of 61 NW (51% boys, BMI: 15.7 ± 1.5 kg/m^2^, 8.5 ± 1.4 yrs) and 59 OW children (47% boys, BMI: 24.2 ± 4.8 kg/m^2^, 8.6 ± 1.4 yrs). For each participant informed consent was signed by both the parents and the school staff. Approval for this study was obtained from the ethics committee of Ghent University Hospital.

### Measurements

Data were collected over a 5 month period from November 2006 to March 2007 (winter time).

#### Anthropometric measurements

Stature was measured to the nearest 0.1 cm using a stadiometer (Holtain Ltd, Crymmych, Pembs, UK). Body mass was measured to the nearest 0.1 kg on a digital balance scale (Seca, max 200 kg, Hamburg, Germany) with the subject wearing lightweight clothing and no shoes. BMI was calculated from height and weight measures. BMI z-scores were calculated based on Flemish reference data [28] using the LMS method [29,30]. BMI z-scores provide a relative measure of adiposity adjusted for age. It is the number of standard deviation units that a person's BMI is deviated from a mean or reference value.

#### Accelerometry

PA levels were objectively assessed using the CSA/MTI accelerometers (model 7164, Florida, USA). This accelerometer is small, lightweight and practical for use in children and has been shown to be a valid and reliable tool for the assessment of PA in children [31-33]. Accelerometers were attached to adjustable elastic belts and worn above the right hipbone, underneath the clothes. Verbal and written instructions for care and placement of the monitor were provided for the parents and children. Children were requested to wear the accelerometer during waking hours for a continuous period of seven days, removing the monitor only for water-based activities and bathing. The accelerometers were set to measure activity counts in an epoch time of one minute. Activity counts are the summation of the accelerations measured over the epoch. Activity data were stored in the memory of the accelerometer and then downloaded to a computer before analysis. A Microsoft Excel-based Macro was used for data reduction and further analyses (MAHUffe Analyzer version 1.9.0.3) (; Ekelund et al.). Days with less than 600 min (or 10 hours) registered time and persons with less than 3 valid days including 1 weekend day were excluded from further analyses. This activity monitoring period is sufficient to obtain reliable estimates of children's' habitual PA [34,35].

Non-wearing time was defined as 60 minutes or more of consecutive zero counts. The outcome variables were total daily counts, counts per minute (cpm) and time spent at activities of different intensity (min/day). Published cut-offs to determine time spent in activities of different intensities vary substantially between studies. In this study sedentary activity was defined as less than 500 counts, low intensity activity as 501 to 2000 counts and moderate to vigorous activity (MVPA) as more than 2000 counts. These cut-offs have been used in the European Youth Heart Study [12] in four regions in Europe (Odense, Denmark; the island of Madeira, Portugal; Oslo, Norway and Tartu, Estonia) and are comparable to the age-specific cut-offs of Freedson for 8.5-yr-old children [36]. According to the prediction equation of Freedson, activities of 4 METs correspond to 1703 counts/min. The chosen cut-off of 2000 counts/min for MVPA is equivalent to walking at 3–4 km/h [37,38]. Weekly number of 5, 10 and 20 minute bouts of MVPA were also calculated. Finally, the proportion of children achieving current health-related PA recommendations of 60 minutes of MVPA per day [39] was estimated.

#### Questionnaire

Parents were asked to fill in a questionnaire on PA and sedentary behaviour and PA related personality of their child. Self-reported weight and height of parents was also requested. Questions regarding PA and sedentary behaviour were adopted from the validated Computerised Flemish Physical Activity Questionnaire [40] and consisted items regarding sport participation, outside play, screen behaviour and inactive play (such as sedentary play, drawing, painting, reading...). There were moderate correlations between items of the questionnaire and the accelerometry data in this study (r between 0.22 and 0.45). Questions regarding PA related personality were developed by the research team based on clinical experiences with OW and obese children. As there was high inter-parental agreement (ICC's ranging from 0.79 to 0.88) for these PA related personality items, mean scores of both parents were used for further analyses. As there was high inter-item agreement (Cronbach's alpha = 0.88, 9 items) a total PA related personality score was calculated.

Research staff was trained in assessment of anthropometric measurements, placement of the accelerometers and providing instructions to parents regarding use of the accelerometers and filling in the questionnaire.

### Statistical analyses

Data were analysed using SPSS software (version 15.0). Values of p < .05 were considered statistically significant. Independent Samples T-tests were used to compare NW and OW children. Pearson Chi-squares were used to investigate the relationship between overweight status (NW versus OW) and categorical variables: having overweight parents (yes versus no), participating in sports (yes versus no) or achieving PA recommendations (yes versus no). Differences in activity levels between week and weekend days were analysed using Repeated Measures ANOVA. Results are presented as means ± SD. A hierarchical multiple regression analysis was executed to investigate the variance in BMI z-score explained from PA related variables after correcting for parents' BMI. PA related variables which significantly correlated with BMI z-score where entered in a second block after BMI of mother and father, which were entered in a first block.

## Results

### Compliance with accelerometer measurement protocol

A total of 97 children out of the total sample provided valid accelerometer data that met all inclusion criteria and could be included in further analyses. Of the omitted children, 6 were excluded for failing to achieve at least 3 days of measurement (including one weekend day), 10 for failing to achieve at least 600 minutes of valid recording on each measurement day, 6 because of instrument malfunction and 1 instrument was lost. Excluded participants did not significantly differ from the study population in gender, age or BMI z-score. On average children had six valid days (NW: 6.3 ± 0.9 days, OW: 6.0 ± 1.2 days) and wore the accelerometer for about 13 hours per day (NW: 778.2 ± 47.6 min, OW: 775.1 ± 63.6 min). There were no differences in number of valid days or registered time per day between OW and NW children. To check for instrument reactivity, the results from day 1 were compared with the mean results of the next days. As activity levels on the first monitoring day did not differ from the next days, all days were included in the analyses.

### Accelerometer data

**Figure 1:**
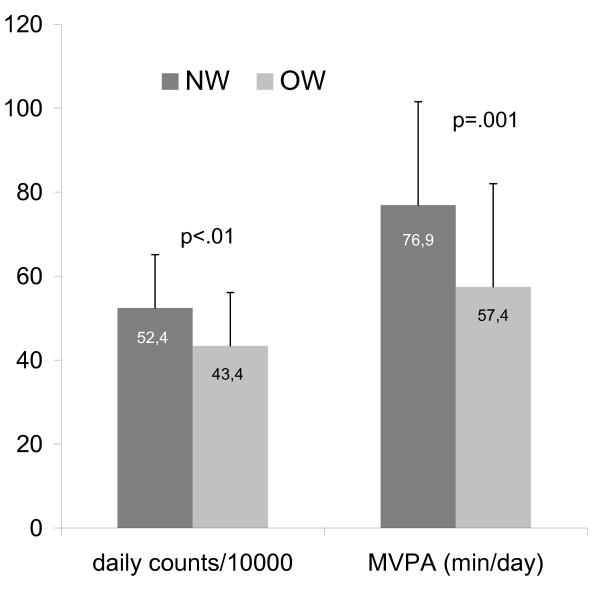
**Degree of physical activity in NW and OW children**.

**Figure 2:**
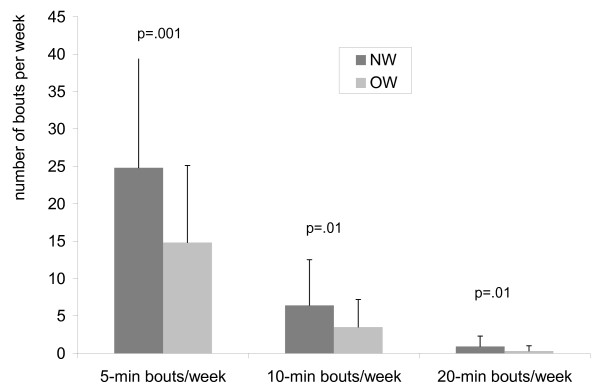
**Number of 5-min, 10-min and 20-min bouts of MVPA in NW and OW children**.

As displayed in Figure 1, OW children had lower mean total daily counts (OW: 556 ± 159 cpm, NW: 677 ± 225 cpm; t = 2.9, p < .01) and lower mean engagement in MVPA (OW: 57 ± 25 min/day, NW: 77 ± 31 min/day; t = 3.3, p = .001) compared to NW children. OW children had also less bouts of 5 minutes (t = 3.6, p = .001), 10 minutes (t = 2.8, p = .01) and 20 minutes (t = 2.8, p = .01) of MVPA (see Figure 2). There was no differences in amount of time spent in light intensity activity (OW: 172 ± 46 min/day, NW: 176 ± 30 min/day; t = 0.5, ns) or sedentary activity (OW: 545 ± 72 min/day, NW: 527 ± 66 min/day; t = -1.3, ns) between both groups.

**Figure 3:**
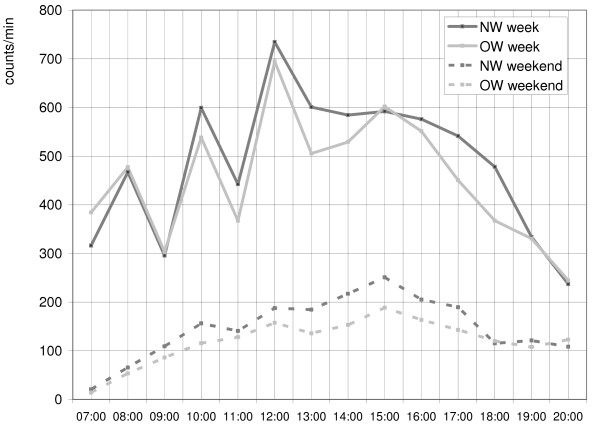
**Physical activity patterns for week and weekend days in NW and OW children**.

Figure 3 shows the PA patterns during the day, separately for week and weekend days in both groups. Periods before 7 am and after 8 pm are not presented as in the majority of children no activity was recorded at these times. OW and NW children had similar patterns of PA, with peaks on week days around recess in the morning and lunch break. Biggest differences between the OW and NW children were in the early afternoon and after school. On weekend days both NW and OW children had very low activity levels with the biggest differences in the afternoon. In both groups activity levels were much higher on week days compared to weekend days (F = 534.2, p < .001).

The recommended activity guideline of 60 minutes of MVPA per day was met by a greater proportion of NW children (71%) compared to OW children (50%) (chi^2 ^= 4.0, p < .05).

### Parents' questionnaire

OW children were more likely to have an overweight (BMI > 25 kg/m^2^) father (chi^2 ^= 6.8, p < .01) or mother (chi^2 ^= 17.9, p < .001) compared to NW children. In 44% of OW children, both parents were overweight, whereas only 8% of NW children had two overweight parents (chi^2 ^= 18.8, p < .001).

A greater proportion of OW children was not engaged in any sport (46% versus 23%, chi^2 ^= 6.3, p = .01). There were no differences in hours of outside play on week days (NW: 0.9 ± 0.9, OW: 0.9 ± 1.2; t = 0.3, n.s.) and weekend days (NW: 2.6 ± 1.5, OW: 2.3 ± 1.7; t = 0.9, n.s). Parents of both OW and NW children reported their child to play outside for about 1 hour on week days and 2.5 hours on weekend days. There were not much differences in sedentary behaviour. Amount of inactive play did not differ between groups on both week days (NW: 1.9 ± 1.5, OW: 1.8 ± 1.3; t = 0.1, n.s.) and weekend days (NW: 2.5 ± 1.6, OW: 2.5 ± 1.5; t = 0.03, n.s.). Screen behaviour on week days was comparable in NW (1.8 ± 1.4 hours/day) and OW (2.2 ± 1.3 hours/day) children (t = -1.5, n.s.), whereas on weekend days OW children showed more screen behaviour (NW: 2.7 ± 1.3, OW: 3.5 ± 1.6; t = -2.7, p < .01). Screen behaviour was higher on weekends compared to week days in both NW and OW children (F = 119.7, p < .001), with bigger differences in OW compared to NW children (F_time × group _= 4.1, p < .05). On week days, screen behaviour exceeded the recommended limit of 2 hours per day in 18% of NW and 33% of OW children (chi^2 ^= 3.0, p = .08). On weekend days, the proportion of children who showed screen behaviour above the recommended limit of 2 hours was 50% in NW children and 67% in OW children (chi^2 ^= 3.6, p = .06).

Results regarding PA related personality of the child as reported by the parents are presented in Table 1. Parents of OW children agree more that their child is born tired, moves slowly, is often tired, lacks energy, avoids physical efforts and prefers watching playing children instead of joining them. Parents of OW children agree less that their child is always active, needs to let himself/herself go and has a lot of energy. Overall, parents of OW children report their child to have a less active personality compared to parents of NW children (t = 5.2, p < .001).

**Table 1:** PA related personality in NW and OW children

	NW	OW	t-value
Born tired° (-)	2.0 ± 1.2	2.5 ± 1.2	-2.1*

Moves slowly° (-)	1.4 ± 0.8	2.5 ± 1.3	-5.5***

Is often tired° (-)	1.9 ± 0.9	2.5 ± 1.3	-2.6**

Lacks energy° (-)	1.5 ± 0.9	2.3 ± 1.4	-3.7***

Avoids physical efforts°(-)	1.6 ± 0.9	2.5 ± 1.2	-4.3***

Prefers watching playing children instead of joining them° (-)	1.5 ± 1.0	2.0 ± 1.2	-2.4**

Is always active° (+)	3.7 ± 1.0	3.0 ± 1.1	2.7**

Needs to let himself/herself go° (+)	4.0 ± 1.0	3.5 ± 0.9	2.8**

Has a lot of energy° (+)	4.0 ± 1.0	3.5 ± 1.3	2.5**

TOTAL SCORE° (+)	4.2 ± 0.7	3.5 ± 0.8	5.2***

°5-point scale (not true at all to very true)

*p < .05

**p < .01

***p < .001

### Regression analysis

Finally, a hierarchical regression analysis was executed to investigate the variance in BMI z-score explained from PA related variables after correcting for parents' BMI (Table 2). PA related variables which correlated signicantly with BMI z-scores were included in the model. The only variables which significantly correlated with BMI z-score were PA related personality (r = -0.42, p < .001), MVPA (r = -0.27, p < .01) and weekend screen behaviour (r = 0.23, p = .01). Intercorrelations between these independent variables were around 0.30 (MVPA-PA personality: r = 0.29, p < 0.01; MVPA-TV: r = -0.29, p < .01; PA personality-TV: r: -0.31, p = .001). Only PA related personality significantly contributed to the explained variance in BMI z-score. Parental BMI explained 29% of the variance in BMI z-score of the child (p < .001) and PA related variables added another 12% of the explained variance (p < .01).

**Table 2:** Variance in BMI z-score explained from PA related variables after correcting for parents' BMI

Dependent variable	Step	Independent variables	Stand. Coeff. Beta	ΔR^2^	Adj R^2^
BMI z-score	1	BMI mother	0.39(***)	0.29(***)	0.27
		BMI father	0.25(*)		
	
	2	PA personality	-0.27(**)	0.12(**)	0.36
		MVPA	-0.06		
		Screen behaviour (weekend)	0.12		

*p < .05

**p < .01

***p < .001

## Discussion

The first purpose of this study was to compare PA and sedentary behaviour in OW and NW 6- to 10-yr-old children. We found that OW children spent on average 20 fewer minutes per day in MVPA (defined as above 2000 cpm) compared to NW counterparts. A recent systematic review of the evidence relating PA to health concluded that children should spend at least 60 minutes in MVPA each day, in order to promote a broad range of health improvements [41]. In the present study, a greater proportion of NW children (71%) compared to OW children (50%) accumulated on average 60 minutes of MVPA per day.

The engagement in MVPA and sedentary behaviour is widely dependent on the cut-point applied to the data [42]. To be able to compare our data with results from other European countries, we used the same cutoff points as in the European Youth Heart Study [12]. Participation in MVPA was lower and sedentary time was higher in our sample of Flemish 6- to 10-yr-old children compared to a representative sample of 9- to 10-yr-old children from Portugal, Denmark, Norway and Estonia. In that European sample, no difference was found in mean fraction of time spent in MVPA between NW and OW children, but obese children were significantly less active. When expressing activity level in cpm, other studies in Europe and the US [13,14,43] found similar differences between NW and OW and/or obese young children as in the present study.

Frequent sustained bouts of MVPA have beneficial effects on a range of cardiovascular risk factors in adults [44]. However, in this study few children achieved sustained bouts, and amount of 5 min, 10 min and 20 min bouts of MVPA per week was lower in OW compared to NW children. Previous studies [45,46] showed that the natural tempo of children's activity is characterised by frequent short bursts of activity lasting just seconds. However, it is unclear what the impact is of these short bursts of activity on children's health. The results of our study are comparable with the findings of Trost et al. [36] who found that 11-yr-old US obese children participated in fewer continuous bouts of MVPA compared to non-obese counterparts. Considering the lower amount of sustained bouts of PA in OW versus NW children and the importance of sustained duration of exercises to promote a significant fat oxidation, it might be important to promote activities of moderate intensity (which can be easily sustained for longer periods) for the primary and secondary prevention of overweight and obesity in children.

We also studied PA patterns during the day, separately for week and weekend days. OW and NW children showed identical PA patterns on both week days and weekends, although at different levels. On week days, both groups showed peaks in PA around recess and lunch break and activity levels tailed off towards the evening. Greatest differences in PA between OW and NW children were found in the afternoon after school. This is in agreement with previous findings that the least active children are especially less active during 'after school hours' [19] and that differences in activity level according to weight status are most marked when children are more likely to be able to choose to be active [13]. In weekends, activity patterns were flatter, without the marked peaks and troughs seen on week days, probably due to the lack of the school structure and consequently the higher diversity of PA patterns. We found much lower activity levels in both OW and NW children on weekends compared to week days. Previous studies also found lower activity levels on weekends in children [45,47]. Consequently interventions with parents are needed to increase activity levels in the home environment during weekends and after school hours. The structure of the day provided by the school appears to be critical to children's PA. The school environment, with time spent in recess play and involvement in physical education classes, provides children opportunities to be PA.

Since accelerometry provides no information about which activities are being carried out at any time, we combined accelerometer-derived data with information about PA behaviour of the child derived from the parents. According to parental reports, OW children had less sport participation, similar outside and inactive play and more screen behaviour on weekends compared to NW counterparts. A previous study also showed that regular participation in at least 3 hours per week of sports activities was associated with lower body fat mass [48]. To our knowledge, no previous studies compared amount of outside or inactive play in OW and NW children. Many previous studies confirm our finding that OW children have more screen behaviour compared to NW children [49,50]. It is recommended that screen behaviour should be limited to no more than two hours per day [51]. On week days, screen behaviour exceeding the recommended limit of 2 hours per day in 18% of NW and 33% of OW children. On weekend days, the proportion of children who showed screen behaviour above the recommended limit of 2 hours was 50% in NW children and 67% in OW children.

Next, we wanted to investigate whether PA related personality differed in OW and NW 6- to 10-yr-old children. The current finding of distinct PA related personality traits in OW children has not been previously reported. Due to the cross-sectional nature of this study, we cannot explore whether OW children have excessive fat mass due to their less active personality or whether their less active personality traits are a consequence of their overweight status. It is also possible that some PA personality traits relate to causes, while others are a result of the overweight status. We do not know either whether this PA personality is inborn and stable across time, or whether PA personality traits may develop or mature over time. However, in a review of the existing literature on genetic determinants of sport participation and daily physical activity, Beunen and Thomis [52] concluded that activity as a personality trait is under genetic control and that no significant shared environment influences were found. A recent study investigating personality continuity in children, found that "energy" personality ("bubbles with life") is stable over time and that continuity was mainly explained by genetic and non-shared environmental factors [53]. Personality theory proposes that personality traits are determinants of physical activity behaviour [54-62]. The effects of personality on physical activity behaviour are thought to be accounted for within a health related theoretical framework. An inactive personality may have a negative impact on social cognitions (attitudes, norms and self-efficacy) towards physical activity, which in turn could influence the activity behaviour itself [54-60]. So, an inborn and stable inactive personality trait may be one reason behind the difficulty to convince overweight and obese people to adopt and especially maintain an active lifestyle. On the other hand, an active personality or an inborn drive to be active may have a positive impact on social cognitions and activity behaviour and may prevent children from developing overweight or obesity.

We also have to consider that it is possible that parental report of the child's PA personality is influenced by weight status of the child and is rather a reflection of their own thoughts about their child than an expression of the child's true PA personality. However, parents may be considered the best informants on their children's behaviour, as they know them best and have the opportunity to observe them in a wide variety of settings. Previous studies showed that a child's personality can be adequately assessed using parental ratings [22-24].

The final purpose of this study was to investigate how much of the variance in overweight status could be explained by PA and PA related personality in 6- to 10-yr-old children. As overweight status of the child is usually associated with parental weight status [63,64], BMI of both parents was first included in the regression model. These variables explained 29% of the variance in the child's overweight status. Obtained correlations between parents' and child's weight status were somewhat higher in magnitude to those observed in prior research [63-65]. PA related variables (MVPA, weekend screen behaviour and PA related personality) added 12% of the explained variance in child's BMI z-score. However, PA personality was the only variable which significantly contributed to the explained variance in overweight status. We found significant correlations between BMI z-score and MVPA (r = -0.27) and weekend screen behaviour (r = 0.23), but these variables did not significantly contribute to the explained variance in overweight status. This is in contrast with the general belief that the current obesity epidemic is mainly due to decreased activity levels and/or increased sedentarity [66,67]. Results from the European Youth Heart Study indicated that less than 1% of the variation in body fatness was explained by time devoted to PA [12]. PA personality may be associated with certain components of activity behaviour or energy expenditure that are not well captured by accelerometry. The correlation between MVPA and PA personality was only 0.29, demonstrating that PA personality and accelerometer-derived MVPA reflect different aspects of activity behaviour. Having an active PA personality may children make less prone to weight gain. Therefore PA related personality may be an important construct in the study of overweight and obesity. However, longitudinal studies are necessary to determine whether PA personality can help identify children at risk for developing overweight. Further studies are needed to explore the role of PA related personality in the development of overweight and obesity.

This study has several limitations. Although accelerometers are the best tool to measure free-living PA in young children, these instruments also have some limitations. Firstly, accelerometers underestimate the activity level during cycling, upper body exercises, walking uphill and stair climbing and cannot be used during water activities. Secondly, as already mentioned above, comparsion in accelerometer-derived MVPA and sedentary behaviour across studies is somewhat difficult because the cutoffs used to define activities of different intensities differ. Moreover, intensity cutoffs have been validated in children, but not specifically in OW children. It may be that the association between movement counts and intensity varies by weight status. Thirdly, the fact that the child wears an accelerometer may modify the child's habitual PA. However, analysis of the mean counts on the first and the next days of recording suggests no systematic differences. But although we controlled for reactivy, it is also possible that children modified their activity behaviour during the total time they were monitoring their behaviour. Fourthly, it is widely believed that epochs shorter than 1 minute would be more appropriate in children because of the perception that children's patterns of PA are highly intermittent. Long epochs of 1 minute as used in this study might indeed misclassify high intensity activity as moderate activity in children [43]. But as we used MVPA, this misclassification is not a real limitation. However, it could be that short bursts of activity are more related to PA personality and/or overweight status in young children. The weak relation between body fatness and MVPA in the European Youth Heart Study [12] might also be explained by the longer epoch of 1 minute used in that study. Next, as this was the first study to investigate PA related personality traits in OW versus NW children, more research is needed to define the construct of PA personality, to investigate which specific traits are part of this construct and to study the stability of this construct over time. Finally, due to the cross-sectional nature of the study, no cause-effect conclusions could be drawn.

## Conclusion

The results of this study demonstrate that NW children spent on average 20 min per day more in MVPA than their OW peers. Physical activities were intermittent and were not sustained for long periods of time in both OW and NW children, with lower amounts of continuous bouts of MVPA in OW children. PA patterns over the day were similar in NW versus OW children, with much lower activity levels on weekends compared to week days. However, clear differences according to weight status were found in the afternoon, with lower activity levels in OW children during "after school hours". This is the first study to show distinct PA related personality traits in OW children compared to NW peers. The results of this study show that measurement of PA personality may help to capture aspects of PA behaviour which are not detected by accelerometry.

## Competing interests

The authors declare that they have no competing interests.

## Authors' contributions

BD, IDB and GC conceived the study. ED was responsible for the data collection. BD participated in its design and coordination, did the statistical analyses and wrote the manuscript. IDB, ED and GC critically reviewed the manuscript for writing and intellectual content. All authors read and approved the final manuscript.
